# Molecular elements: novel approaches for molecular building

**DOI:** 10.1098/rstb.2022.0024

**Published:** 2023-02-27

**Authors:** Ruowen Wang, Xueqiang Wang, Sitao Xie, Yanyan Zhang, Dingkun Ji, Xiaobing Zhang, Cheng Cui, Jianhui Jiang, Weihong Tan

**Affiliations:** ^1^ Institute of Molecular Medicine (IMM), Renji Hospital, School of Medicine, School of Chemistry and Chemical Engineering, Shanghai Jiao Tong University, Shanghai 200127, People's Republic of China; ^2^ Zhejiang Cancer Hospital, Hangzhou Institute of Medicine (HIM), Hangzhou, Zhejiang 310018, People's Republic of China; ^3^ Molecular Science and Biomedicine Laboratory (MBL), State Key Laboratory of Chemo/Biosensing and Chemometrics, College of Chemistry and Chemical Engineering, College of Biology, and Aptamer Engineering Center of Hunan Province, Hunan University, Changsha, Hunan 410082, People's Republic of China; ^4^ Department of Chemistry, Department of Physiology and Functional Genomics, Center for Research at Bio/Nano Interface, Health Cancer Center, University of Florida Genetics Institute and McKnight Brain Institute, University of Florida, Gainesville, FL 32611-7200, USA

**Keywords:** molecular element, oligonucleotide, nucleic acid therapeutics, aptamer, unnatural base

## Abstract

Classically, a molecular element (ME) is a pure substance composed of two or more atoms of the same element. However, MEs, in the context of this review, can be any molecules as elements bonded together into the backbone of synthetic oligonucleotides (ONs) with designed sequences and functions, including natural A, T, C, G, U, and unnatural bases. The use of MEs can facilitate the synthesis of designer molecules and smart materials. In particular, we discuss the landmarks associated with DNA structure and related technologies, as well as the extensive application of ONs, the ideal type of molecules for intervention therapy aimed at correcting disease-causing genetic errors (indels). It is herein concluded that the discovery of ON therapeutics and the fabrication of designer molecules or nanostructures depend on the ME concept that we previously published. Accordingly, ME will be our focal point as we discuss related research directions and perspectives in making molecules and materials.

This article is part of the theme issue ‘Reactivity and mechanism in chemical and synthetic biology’.

## 1. Introduction

**Figure 1.**
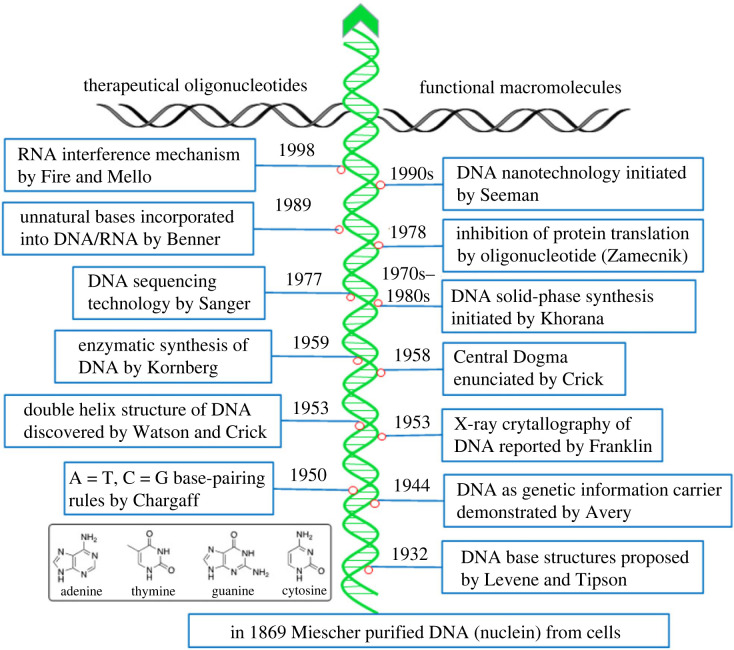
Landmarks associated with DNA structure and technologies. (Online version in colour.)

DNA is the molecular foundation of the biological system as it is the genetic information carrier stored in the nucleus [1]. Compared with other biomacromolecules, such as proteins and polysaccharides, the structure of DNA is much simpler since it is composed of only four types of units. The evolution of DNA structure and biology is the central part of science, having produced many revolutionary technologies changing the lifestyle of human beings (figure 1). Although DNA was discovered as a natural chemical from living systems by Miescher in 1869 [2], the ring structures of ATCG bases were not identified by Levene and Tipson until 1932 [3,4]. The role of DNA as a gene information carrier in the cell was proposed by Oswald Avery in 1944 for the first time [5,6]. Shortly after the establishment of A = T and C = G base-pairing rules by Chargaff [7], Watson and Crick discovered the double-helix structure of DNA inspired by Franklin's X-ray crystallography of DNA in 1953 [8–11]. The start of molecular biology signalled a breakthrough in genomics and thereafter spurred landmark discoveries in science and technological advances (figure 1).

Molecular biology, as a new field, started from the discovery of the DNA duplex structure, which had not been fully established until Crick *et al*. enunciated their ‘Central Dogma’ [12,13] and deciphered the genetic code [14]. In the words of Crick, nearly ‘all aspects of life are engineered at the molecular level, and without understanding molecules, we can only have a very sketchy understanding of life itself’ (see https://profiles.nlm.nih.gov/spotlight/sc/feature/biographical-overview). Indeed, Crick's opinion is supported by ever more scientific discoveries. For example, the discovery of DNA polymerase by Kornberg [15,16] led to the establishment of enzymatic synthesis technology [17] and DNA sequencing [18,19] upon which modern biotechnology is based. DNA sequencing initiated by Sanger enables gene sequencing accessible to the general public. With genetic information, we can understand diseases at the molecular level and find cures.

Designable nucleic acids are unique probes for biological studies. Many structure–function studies have resulted in the efficient preparation of oligonucleotides (ONs) ever since Khorana managed to chemically synthesize a gene in the laboratory for the first time [20,21]. Aided by ON synthesis technology, the Dickerson and Rich groups reported the crystal structures of A-, B- and Z-DNA fragments [22,23], which is important because the helical structure of DNA is variable under different environments and closely related to biological properties. Beyond biotechnology, ONs prepared by DNA synthesizers have been extensively applied in materials science [24], nanotechnology [25–27], information technology [28,29] and clinical diagnosis and therapies [30]. Accordingly, many different functionalities have been designed and incorporated into nucleic acids to meet specific requirements [31,32].

## 2. Unnatural DNA bases: bottom-up elements

The fundamental role of DNA and its quite simple structure has long evoked curiosity. For instance, to investigate if ATCG could be replaced by other functionalities, the Benner group designed unnatural bases with close similarity to ATCG bases and realized enzymatic incorporation of unnatural bases into RNA with high accuracy [33,34]. To determine if hydrogen bonding is necessary for DNA base-pairing, the Kool group found that well-designed aromatic functionalities could work as a pair of hydrophobic bases stabilizing the DNA duplex [35,36]. They also designed a series of size-expanded bases from which more thermodynamically stable DNA duplexes were prepared, such as xDNA and yDNA [37–39]. We have designed and synthesized the most size-expanded unnatural base by fusion of an azobenzene with a natural T base to give base zT, which is capable of specific base-pairing with natural A through hydrogen bonding [40]. Unnatural base-pairing was also introduced to the duplex by replacing hydrogen bonding with metal-mediated bonding [41,42]. The Romesberg group designed and synthesized more than a thousand hydrophobic bases out of which they screened some unnatural bases that function in a manner similar to that of ATCG bases in the cell system following ‘Central Dogma’ [43–46].

**Figure 2.**
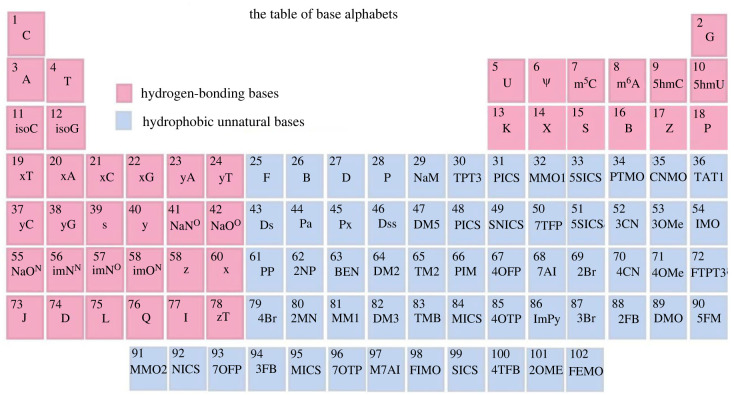
List of base alphabets expressed in periodic table style. (Online version in colour.)

The incorporation of unnatural bases into nucleic acids provides a unique insight into DNA biology and function. In order to distinguish natural AT(U)GC bases from unnatural bases, Benner proposed unnatural bases as DNA's new alphabets [33]. Encouraged by the discovery of Benner's unnatural bases in the 1980s, a few groups have since made major contributions to this field [47–54]. In fact, more than 100 unnatural bases have been reported with base-pairing properties, enough to fill the elemental table (figure 2). Several recently published reviews have described the progress in this field, which we are not discussing in detail here [48,55–57].

It is exciting, but challenging, to reconstruct a life system in a bottom-up approach with unnatural bases. On the other hand, unnatural bases may find unique applications in biotechnology and biomedicine [58]. The Hirao group has created high-affinity DNA aptamers with unnatural bases, which specifically bind to target proteins with improved biostability [59,60]. Our collaboration with the Benner group resulted in the generation of aptamers that selectively bind liver cancer cells. These aptamers evolved from a six-letter DNA library with unnatural bases Z and P [61–63]. It has been demonstrated that DNA is a unique data storage device for information technology (IT) [64]. Accordingly, unnatural bases may find unique functions in IT as data storage and read-out systems. Simpler than cellular systems, the addition of a base ‘byte’ would multiply the capacity of data storage.

## 3. Molecular elements: concept and the significance

The convenience of building ON molecules by automated synthesis has inspired applications of ONs in basic research and clinical medicine. To produce such ONs, nucleoside phosphoramidites, first identified in 1981 [65,66], allow sequential addition of new bases to the DNA chain. More than 1000 phosphoramidites have been reported [67–71], providing infinite possibilities for all kinds of technical nucleic acids, or TcNA, available for functionalization within the scope of the periodic table of elements shown in figure 2. However, the rational design of small molecules with specialized functionalities calls for the development of better guidance to address the demand for new nucleic acid-based materials and nucleic acid-based therapeutics, leading to the expansion of TcNA applications. Accordingly, in 2017, we introduced the concept of the molecular element (ME) [40], which we describe below.

**Figure 3.**
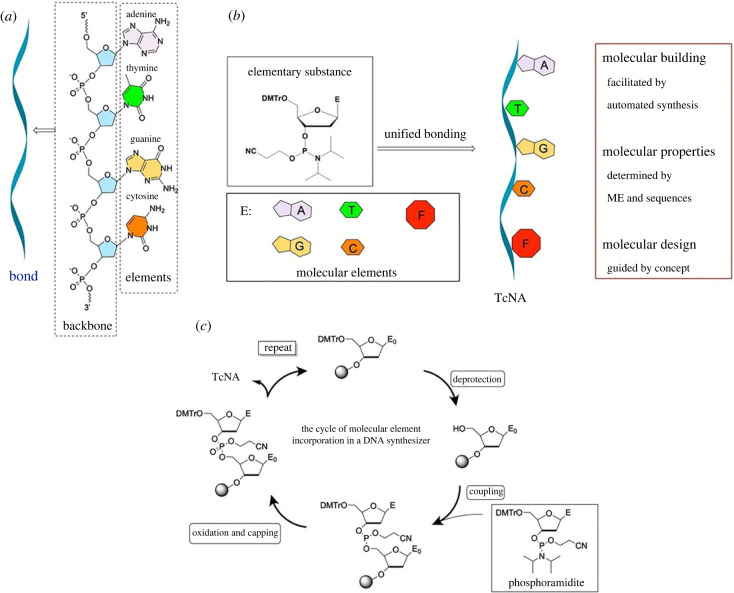
Structure of a single-strand DNA sequence (*a*), the concept of molecular elements (*b*), and a brief introduction of the automated synthesis (*c*). (Online version in colour.)

From the structure of a single-stranded DNA (figure 3*a*), it is obvious that the backbone of the molecule is uniform and that every unit differs from others by base moieties. Hence, the sugar–phosphate–sugar backbone may be abstracted as the ‘bond’ of nucleic acids and the base moiety as the ‘element’. Adenine pairs with thymine, and cytosine pairs with guanine. Based on this simplified DNA single-strand structure, we propose the ME concept.

A ME can be any molecule with special functions, including both natural A, T, C, G, U and unnatural bases (figure 3*b*). MEs are converted to corresponding phosphoramidites by organic synthesis as elementary substances for construction of TcNAs. As shown in figure 3*b*, from phosphoramidites, an individual ME is bonded together into the backbone of ONs during automated synthesis step-by-step in a programmable approach with up to 99.9% yield (figure 3*c*). As demonstrated by nature, the sequences of four MEs, A, T, C and G, store huge genetic information and biological functions. The discovery of more functional MEs will lead to the construction of TcNAs with infinite functions, and people can build their dream molecules as easily as shopping in a molecular supermarket.

Through the programmable assembly of functional moieties onto the DNA backbone, TcNAs can be turned into diagnostic probes, catalytic molecules or therapeutic molecules. Examples of such conjugates include lipid-, polymer- or nanoparticle-DNA. MEs can also guide the construction of nanodevices with diverse functions. More to the point, the confluence of ME and TcNA offers these merits: (i) simple and efficient design of molecular-level constructs in that all MEs are bonded in sequence through the same phosphoramidite chemistry; (ii) infinite molecular properties since the properties of TcNAs are determined by the incorporated MEs and their sequences; and (iii) rational design of TcNAs realized under the ME framework in future when more underlying disciplines are discovered.

## 4. Engineering technical nucleic acids

**Figure 4.**
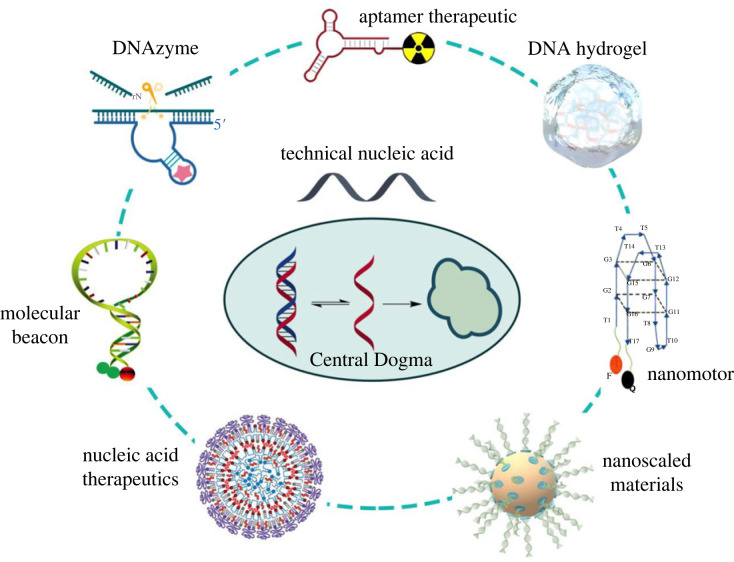
Extensive applications of TcNAs as derivative molecules of DNA beyond ‘Central Dogma’. (Online version in colour.)

The progress in nucleic acid preparations, including both chemical and biological synthesis, has allowed researchers to use nucleic acids as unique tools. TcNAs can be prepared by automated synthesis, and the sequences are programmable. Furthermore, the structure can be readily modified through the incorporation of functional moieties. Commercially available TcNAs have been extensively explored in medicine, chemistry, physics, materials science and even information technology. DNA nanotechnology, DNA-based advanced materials and nucleic acid therapeutics have emerged as the frontiers in interdisciplinary fields (figure 4).

Programmable sequence and specific A-T and C-G base-pairing modality afford researchers the opportunity to construct designer molecules or nano-scaled devices. For example, aptamers are generated from a library of ONs binding to targets with high affinity and specificity, many of which have been developed as therapeutic molecules for clinical applications [72–77]. Molecular beacons, single-stranded ONs with hairpin-loop conformation, are also used in a variety of formats, such as *in vitro* RNA and DNA monitoring, biosensors and real-time monitoring of gene expression in living systems [78–81]. DNAzymes are also single-stranded ONs capable of catalysing chemical reactions as enzymes; DNAzymes have received attention for bioimaging and biosensor development [82–84]. A molecular nanomotor can be constructed by a single-stranded ON, which is fuelled by the hybridization of DNA [85–87]. Hybridization and dehydration between base-pairing are processes which, under programmable control, have been used to prepare intellectual hydrogels, soft nanomotor devices and biomimetic DNA nanostructures [88–94].

DNA has had a remarkable impact on nanoscience and nanotechnology with the most predictable interactions of all molecules. Through specific base-pairing, programmed TcNAs can assemble structural motifs and then connect them, fabricating nano-scaled structures in a bottom-up approach [95–97]. DNA nanotechnology presents the advantages of chemical diversity, highly programmable synthesis and precisely controllable structure, as demonstrated by DNA origami and DNA-mediated nanoparticle assembly [98–100]. When more MEs are introduced into TcNAs besides natural ATCG, more powerful nanodevices can be engineered with functional TcNAs.

## 5. Nucleic acid therapeutics

We are entering an era in which a vast amount of gene sequencing information is available to medical researchers able to take advantage of the completed human genome project, the breakthrough in DNA sequencing technology and large-scale studies of genetic variation.

Originally, molecular medicine involved the application of genetic knowledge to the practice of therapy. Today, a major mission of molecular medicine is to identify pathogenic genetic mutations and develop molecular interventions. Indeed, a major challenge in molecular medicine involves the discovery of molecular therapeutics against disease-causing genetic mutations.

In 1978, it was discovered that the expression of proteins could be disturbed by an exogenous ON complementary to the target gene [101], and the mechanisms of RNA interference were addressed by Mello and Fire in 1998 [102]. Biological experiments have unequivocally verified that both antisense oligonucleotides (ASO) and small interference RNA (siRNA) are powerful and efficient molecular interventions of target genes [30,102–105].

However, it took almost 20 years until the first siRNA was approved for clinical treatment of rare diseases in 2018 [106]. It was a long scientific trek marked by trial and error and continuous structural optimization for therapeutic nucleic acids [31,32,107–111]. Chemically modified ONs have been extensively studied for the development of therapeutic antisense and siRNA because modifications were found to dramatically improve the drug-like properties of ONs, such as cellular uptake, biostability, target specificity and binding affinity. Simple structure, programmable synthesis and ready functionalization are the prominent properties of TcNA, which will benefit clinical applications. However, major challenges arise from the difficulty in delivering TcNA therapeutics to their target tissues. Many efforts have contributed to the development of delivery systems for TcNAs.

Recently, we demonstrated that the structure of ONs can be optimized by incorporating functional MEs using a programmable approach [112]. For delivery systems, these innovations include our lipid nanoparticle (LNP), as well as viral and polymeric delivery systems [113–118]. LNP is the most prominent system, which has been successfully used in the formulation of the first approved siRNA. The formulation of LNP suitable for clinical applications is challenging because the complicated system involves a huge number of chemicals being used as the components. To handle such difficulties, artificial intelligence technology has been applied to the fabrication of LNP [119,120].

## 6. Future directions

### (a) The development of novel molecular elements

Targeted delivery of TcNA therapeutics is very important, as has been demonstrated by the GalNAc platform applied in siRNA functionalization [118]. The development of targeting-ME could enhance the accumulation of TcNA in target tissues and thus improve efficacy. We recently developed a series of microenvironment-targeting MEs and demonstrated that the tumor-specific delivery of ASO is achievable using MEs. Different diseases may vary from each other with a characteristic microenvironment [121–123], which can be used in the design and development of novel MEs.

Pseudouridine (*Ψ*) is an isomer of the nucleoside uridine in which the uracil is attached via a carbon–carbon instead of a nitrogen–carbon glycosidic bond. Its incorporation into messenger RNA (mRNA) enhances translation efficiency [124,125] This ME has been used as an important tool for the development of mRNA therapeutics. Recently, we demonstrated that the structure of ONs can be optimized by incorporating functionalities in a programmable approach [112]. DNA structure is predictable owing to specific base-pairing through hydrogen bonding. While the interaction of TcNA with a serum protein is weak and uncontrollable, the introduction of hydrophobic or cationic functionalities may alter the binding affinity, and change the fate of TcNA in blood circulation [126]. The development of such assembling MEs may provide a sheltering function for TcNAs in the circulatory system.

### (b) Correlations between the sequences of molecular elements and functions

Studies in the structural modification of TcNA have increased through the years. Indeed, over past decades, ON modification has largely broadened the functions and applications of TcNAs and contributed to the clinical success of therapeutic nucleic acids. The advent of phosphoramidite chemistry, as previously noted, has accelerated the functionalization of ONs with modified nucleobases, phosphate-protecting groups and modified sugars [31,32,108,110–114]. Mother Nature has exhibited the power of sequence with four MEs, A, T, C and G, since one single-base mutation results in quite different biological morphology. It will be even more striking and significant to find out how ME sequences change biological properties and discover the existence of synergism between drug payloads and MEs. The discovery of latent principles may provide guidance for molecular design of TcNAs and lead to further breakthroughs.

## Data Availability

This article has no additional data.
